# *Toxoplasma gondii* effector GRA35 mediates neuronal damage *via* ER stress and mitochondria-associated apoptosis

**DOI:** 10.1080/21505594.2026.2654261

**Published:** 2026-04-06

**Authors:** Jie Wang, Ying Chen, Nan Zhou, Fangmin Li, Niuniu Dai, Zhiang Chen, Shutong Liu, Ran An, Lijian Chen, Jian Du

**Affiliations:** a Department of Biochemistry and Molecular Biology, School of Basic Medical Sciences, Anhui Medical University, Hefei, China; b The Provincial Key Laboratory of Zoonoses of High Institutions in Anhui, Anhui Medical University, Hefei, China; c School of Nursing, Anhui Medical University, Hefei, China; d Department of Anesthesiology, The First Affiliated Hospital of Anhui Medical University, Hefei, China

**Keywords:** Toxoplasma gondii, GRA35, RTN1-c, encephalitis, apoptosis

## Abstract

Encephalitis resulting from acute reactivation of chronic *Toxoplasma gondii* infection in the central nervous system poses a significant mortality risk in immunodeficient individuals. However, the specific molecular mechanisms underlying this process remain elusive. We constructed the GRA35 gene knockout ME49 strain and compared the differences with wild type ME49 strain. We used the GST-pull down experiment to explore the mechanism of GRA35 promoting neuronal cell apoptosis. We used immunofluorescence, flow cytometry and CCK8 experiments to verify the pathway of GRA35 promoting neuronal cell apoptosis. Our study reveals that wild type ME49 strain promotes neuronal apoptosis in brain following chronic infection activation. Conversely, infection with the ME49^Δ*gra35*^ strain leads to a reduced apoptotic response in brain neurons. Furthermore, we demonstrate that GRA35 interacts with RTN1-c, thereby promoting mitochondrial pathway-mediated apoptosis in neurons. Additionally, GRA35 can trigger host cell ER stress-associated apoptosis through the PERK signaling pathway. GRA35 serves as a crucial virulence factor in the pathogenesis of Toxoplasmic encephalitis (TE), which offers potential new therapeutic target and theoretical insights for TE.

## Introduction

*Toxoplasma gondii*, an obligate intracellular parasite belonging to the phylum Apicomplexa, infects approximately one-third of the global population [1,2]. Although toxoplasmosis typically only induces mild illness in immunocompetent individuals, it poses significant risks and mortality rates for immunocompromised patients [3]. The primary cause is the acute activation of infection within the central nervous system, leading to the development of TE [4].

Although studies have revealed the association of specific effector molecules with the pathogenesis of TE, its complexity and current clinical treatment challenges underscore the need for further exploration of the underlying mechanisms [5,6].

The pathogenesis of TE primarily involves the induction of neuronal apoptosis by *Toxoplasma gondii* infection, as supported by various studies [7,8]. Apoptosis pathways include the death receptor pathway, mitochondrial pathway, and endoplasmic reticulum (ER) pathway [9–11]. Death receptors, located on the cell surface, selectively bind ligands carrying apoptotic signals to efficiently induce apoptosis [12]. The mitochondrial pathway entails the release of cytochrome c, disrupting electron transport and oxidative phosphorylation, leading to a reduction in ATP production [13]. Upon transmission of apoptotic signals to mitochondria, BAX channels form on the outer membrane, facilitating the discharge of cytochrome c from mitochondria [14]. Anti-apoptotic proteins Bcl-2 and Bcl-XL on the outer mitochondrial membrane inhibit the release of cytochrome c, thereby suppressing mitochondrial pathway-mediated apoptosis [14]. The ER is a ubiquitous cellular organelle that plays a pivotal role in protein synthesis, folding, and assembly [15]. The excessive accumulation of unfolded and misfolded proteins, along with perturbations in calcium homeostasis, can induce ER stress [16]. In response to this stress, ER-resident transmembrane signaling proteins activate the unfolded protein response (UPR) to restore proteostasis [16]. The UPR consists of three regulatory pathways governed by three ER receptor proteins: PKR-like ER kinase (PERK), activating transcription factor 6 (ATF6), and inositol-requiring enzyme 1 (IRE1) [17]. Under normal conditions, glucose-regulated protein 78 (GRP78) binds to these three ER receptor proteins. However, during ER stress, GRP78 dissociates from the receptors to initiate the UPR. Prolonged or severe ER stress can lead to cell death through this mechanism [18].

The recently discovered dense granule protein GRA35 localizes on the PV membrane and is implicated in the activation of the NLRP1 inflammasome, leading to pyroptosis in Lewis rat macrophages [19]. In this study, we observed a higher expression of GRA35 in type II strains ME49 and Chinese 1 Wh6 compared to type I strains RH and Chinese 1 Wh3. Using CRISPR-Cas9 technology, we successfully generated a GRA35-knockout ME49 (ME49^Δ*gra35*^) strain and demonstrated that GRA35 promoted apoptosis in neurons. By investigating the impact of GRA35 on neuronal apoptosis and elucidating its role in *Toxoplasma* encephalopathy, we aim to provide novel theoretical and experimental evidence for the prevention and treatment of TE, thereby enhancing our understanding of this disease.

## Materials and methods

### Cell culture and treatment

Neuro2a cells were purchased from ATCC, cultured in DMEM supplemented with 10% fetal bovine serum, and maintained in a 5% CO_2_ atmosphere at 37 °C. Subsequently, the cells were seeded into a six-well plate at a density of 1 × 10^6^ cells per well and incubated overnight at 37 °C. To induce neuronal differentiation, the Neuro2a cells were then cultured for an additional 24 h in DMEM with 2% FBS and 20 µM retinoic acid (R2625, Sigma) to induce neuronal differentiation [20–22].

### Reagents and antibodies

The PUC19 plasmid and the pSAG1:CAS9-U6:sgUPRT plasmid were kindly donated by Professor Shen Bang of Huazhong Agricultural University. The sentinel mutation kit(Catalog #: E0552S) was purchased from NEB. Multi-fragment one-step ligation kit(Catalog #: C113-01) was purchased from Vazyme. DBA-FITC(Catalog #: L32474) was purchased from Invitrogen. CCK8 kit(Catalog #: C0037) was purchased from Beyotime. Apoptosis flow assay kit(Catalog #: 560,930) purchased from BD. Mitochondrial membrane potential assay kit(Catalog #: C1071S) and mitochondrial protein isolation kit(Catalog #: C3601) were purchased from Beyotime. NeuN mouse monoclonal antibody(Catalog #: HA601111) purchased from HuaBio. Cyt-c rabbit polyclonal antibody(Catalog #: WL02410), COX IV rabbit polyclonal antibody(Catalog #: WL02203) purchased from Wanlei Biologicals. GAPDH mouse monoclonal antibody(Catalog #: 60,004–1-Ig), HRP-conjugated GAPDH monoclonal antibody(Catalog #: HRP-60004), RTN1-c rabbit polyclonal antibody(Catalog #: 15,048–1-AP), Caspase-12 Rabbit polyclonal antibody(Catalog #: 55,238–1-AP) and Cleaved Caspase-3 rabbit polyclonal antibody(Catalog #: 25,128–1-AP) were purchased from proteintech. HA-tag mouse monoclonal antibody(Catalog #: T0008) were purchased from affinity. GRA35 rabbit polyclonal antibody were produced in the laboratory. Bcl-XL rabbit polyclonal antibody(Catalog #: 2764S), PERK rabbit polyclonal antibody (Catalog #: 5683S), p-PERK rabbit polyclonal antibody(Catalog #: 3179S), p-eIF2α rabbit polyclonal antibody(Catalog #: 9721S), ATF4 rabbit polyclonal antibody(Catalog #: 11815S), GRP78 rabbit polyclonal antibody(Catalog #: 3177S), CHOP rabbit polyclonal antibody (Catalog #: 5554S) all purchased from Cell Signaling Technology. Multicolor prestained protein ladder (Catalog #: WJ103) purchased from Epizyme. GSK2606414(Catalog #: HY-18072), TUDCA(Catalog #: HY-19696), Tunicamycin(Catalog #: HY-A0098) all purchased from MCE.

### Animals and *Toxoplasma gondii*

The C57BL/6 and *Balb/c* mice, both female and aged 6–8 weeks, were procured from the Experimental Animal Centre of Anhui Medical University. After being anesthetized by inhaling 3% isoflurane (RWD Life Science, Shenzhen, China), the mice were placed in a supine position on the operating table. Subsequently, the concentration of isoflurane was adjusted to 1.5% to maintain the anesthetic state. Thereafter, cardiac perfusion was carried out, and the brains were harvested. All *Toxoplasma gondii* strains utilized in this study were maintained through laboratory passaging.

### Plasmid construction

Mutate the pSAG1:CAS9-U6:sgUPRT plasmid to pSAG1:CAS9-U6:SgGRA35 plasmid according to the instructions of the sentinel mutation kit. The donor plasmid was constructed by inserting 1kb upstream of the CDS region of the GRA35 gene (GRA35-up), DHFR-TS, and 1kb downstream of the CDS region of the GRA35 gene (GRA35-down) into PUC19 in sequence using a multifragment one-step ligation kit. Subsequently, PCR amplification was performed to obtain the donor fragment spanning from GRA35-up to GRA35-down. The primer sequences are presented in the Supplementary data (Table S1). The pCMV-Myc-GRA35-HA plasmid was generated by incorporating the GRA35-HA fragment into the multiclonal site of the pCMV-Myc plasmid vector utilizing the restriction endonucleases EcoRI and XhoI. The primer sequences are presented in the Supplementary data (Table S2).

### Generation of parasite strains

The pSAG1:CAS9-U6:sgGRA35 plasmid (25 μg) was mixed with the donor fragment (5 μg), with ATP_2_ Na_2_ (1 mM), and GSH (2.5 mM) into a 300 μl system. 2 × 10^7^ ME49 wild type strains were added to the system and the culture was continued after 1600 V, 25 μF electroshock. The ME49^Δ*gra*35^ strain was selected by adding 5 μM pyrimethamine to the *Toxoplasma gondii* culture medium.

### Mice survival experiments

*Balb/c* mice were randomly allocated into a high-dose group and a low-dose group. In the high-dose group, 12 mice were selected at random and intraperitoneally injected with 1 × 10^6^ ME49^WT^ strains each; while another set of 12 mice were randomly chosen and similarly injected with 1 × 10^6^ ME49^Δ*gra35*^ strains. For the low-dose group, we randomly selected 12 mice for intraperitoneal injection of 1 × 10^2^ ME49^WT^ strains each [23]; whereas another set of 12 mice were also randomly chosen for injection with an equal dose of ME49^Δ*gra35*^ strains. The survival of test mice was monitored for a period of up to thirty days or until all animals had perished. Serum samples were collected from the surviving mice, and seronegative animals were not included in the subsequent statistical analysis [24].

### *Toxoplasma gondii* proliferation assay

Inoculation of 1 × 10^4^ HFF cells into a 24-well plate with a 14 mm cell glass slide attached to the bottom. After the HFF cells grew normally against the wall, the ME49^WT^ and ME49^Δ*gra35*^ strains were added (MOI = 3) and incubated for 24 h. Discard the medium, wash three times with PBS to wash off excess impurities and fix in 4% paraformaldehyde for 30 minutes. Washed three times with PBS to remove residual paraformaldehyde. The cells were stained with Giemsa stain at room temperature for 30 minutes and then rinsed slowly with distilled water. Finally, the proliferation of tachyzoites in HFF cells was observed under a microscope and the number of tachyzoites was counted.

### *Toxoplasma gondii* cyst formation assay

The C57BL/6 mice were randomly divided into ME49^WT^ and ME49^Δ*gra35*^ groups and each group was injected intraperitoneally with 1 × 10^3^ *Toxoplasma gondii* per mouse and continued to be reared for 2 months. Mice were euthanized, brain tissue was collected and ground into a homogenate and the cysts were stained with DAB-FITC staining solution(Invitrogen, California, USA). Microscopic observation to count the number of brain cysts.

### Induction of acute *Toxoplasma gondii* encephalopathy in mice

The C57BL/6 mice were randomly divided into ME49^WT^ and ME49^Δ*gra35*^ groups and each group was injected intraperitoneally with 1 × 10^3^ *Toxoplasma gondii* per mouse and continued to be reared for 2 months. Intraperitoneal injection of 50 mg/kg cyclophosphamide injection once daily for 7 d. Mice were euthanized, brain tissue was collected, fixed, embedded, sectioned and stained [25].

### Immunoblotting

After cellular protein extraction, the proteins were denatured by boiling at 100 °C for 10 min and added to 12% SDS-PAGE gel in electrophoresis to separate the individual molecular weights. The proteins separated on SDS-PAGE were transferred to PVDF membranes and incubated with the corresponding primary and secondary antibodies respectively. Development using ECL, analysis.

### Cell activity assay

1 × 10^3^ Neuro2a cells were grown in each well of a 96-well plate, and ME49^WT^ and ME49^Δ*gra35*^ strains (MOI = 3) were added separately when the cells entered the logarithmic growth phase and incubated for 36 h. Replace the medium with serum-free DMEM, add 10 μl CCK8 reagent to each well, incubate for 1–4 h at 37 °C, protected from light, and read the absorbance at 450 nm.

### Flow cytometry

1 × 10^5^ Neuro2a cells were seeded in each well of a 6-well plate and the ME49^WT^ strain and ME49^Δ*gra35*^ strain (MOI = 3) were added separately when the cells entered the logarithmic growth phase and incubated for 36 h. The cells were collected from each well, stained with PE Annexin V kit, and the cells were collected using CytExpert analytical flow cytometry to analyze the apoptosis of each group of cells.

### Mitochondrial membrane potential detection

1 × 10^5^ Neuro2a cells were seeded in each well of a 6-well plate and the ME49^WT^ strain and ME49^Δ*gra35*^ strain (MOI = 3) were added separately when the cells entered the logarithmic growth phase and incubated for 36 h. The groups of cells were stained using the Mitochondrial Membrane Potential Assay Kit and the cells were collected using a CytExpert analytical flow cytometer to analyze the differences in mitochondrial membrane potential between the groups.

### Immunoprecipitation assay

The cells were transfected with pCMV-Myc-GRA35-HA plasmid and pCMV-Myc plasmid respectively at logarithmic growth stage, and the cells were collected and lysed with IP lysis buffer (0.1% Triton) after 24 h of incubation. The supernatant was added to protein A beads pre-coupled with HA antibody and incubated for 4 h. The protein A beads were washed well and the results were examined by Western blot.

### Mitochondrial protein isolation assay

2 × 10^6^ Neuro2a cells were seeded in 10 cm dishes and the ME49^WT^ strain and ME49^Δ*gra35*^ strain (MOI = 3) were added separately when the cells entered the logarithmic growth phase and the culture was continued for 18 h. Mitochondria were isolated from the cells using the Mitochondrial Extraction Kit (Beyotime, C3601). Mitochondrial proteins were detected separately from cytoplasmic proteins using Western blot. Among them, the marker for mitochondria is COX-V, and the marker for the cytoplasm is GAPDH.

### Immunofluorescence

Inoculate 1 × 10^4^ Neuro2a cells into a 24-well plate with a 14 mm cell glass slide attached to the bottom. After the Neuro2a cells had grown normally against the wall, the ME49^WT^ and ME49^Δ*gra35*^ strains were added (MOI = 3) and incubated for 24 h. Discard excess medium and fix in paraformaldehyde for 15 min. The wells were punched with 0.1% Triton for 5 min and blocked with 5% BSA for 30 min at 37 °C. The wells were incubated with the appropriate primary antibody and secondary antibody, respectively. The smears were stained with DAPI and blocked. The smears were observed and recorded under a laser confocal microscope.

### Statistical analysis

Data are expressed as the mean standard deviation (SD) of three or more independent experiments. One-way analysis of variance (ANOVA) was utilized to compare multiple groups, and two-tailed Student’s t tests were used for two-group comparisons. *p* < 0.05 was considered statistically significant. Analyses were performed using Graphpad Prism (Version 8.02).

## Results

### Construction of GRA35 gene knockout ME49 *Toxoplasma gondii*

**Figure 1.**
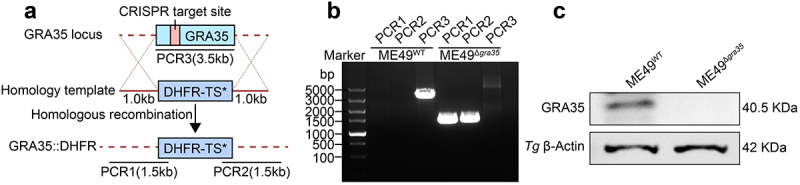
Construction of GRA35 gene knockout ME49 *Toxoplasma gondii*. (a) schematic representation of the *gra35* gene knockout program. *gra35* gene was replaced by a pyrimethamine-resistant DHFR* fragment. (b) PCR detection of *gra35* gene knockout. PCR1 and PCR2 demonstrate successful integration of upstream and downstream homology arms of *gra35* gene; PCR3 demonstrate successful knockout of the *gra35* gene. (c) WB detection of *gra35* gene knockout.

Studies have indicated that human infection primarily occurs with less virulent strains of parasites [26]. Therefore, this study focuses on the ME49 strain of type II. To investigate the role of GRA35 in the interaction between *Toxoplasma gondii* and its host, we utilized CRISPR-Cas9 technology to knockout *gra35* gene in the less virulent ME49 strain (ME49^Δ*gra35*^) and conducted validation experiments (Figure 1(a-c)). The results revealed that the *gra35* gene knockout ME49 strain was successfully constructed.

### GRA35 induces neuronal apoptosis during toxoplasmosis

**Figure 2.**
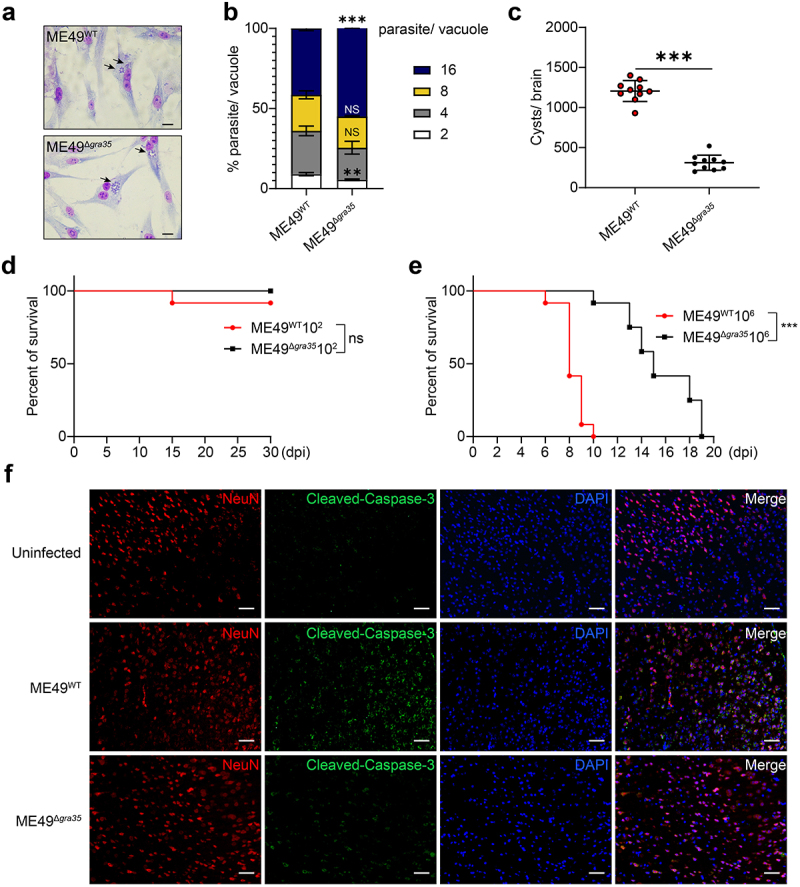
GRA35 promoted neuronal apoptosis in the brain of C57BL/6 mice during TE. (a) Representative image of gimsa staining. Scale bar = 50 μm. (b) The statistical quantification of parasite/vacuole in gimsa staining. means ± SD of three independent experiments; ****p < 0.0001, unpaired t test. (c) Results of the mouse cyst formation assay. (d) Survival curves after infection of *Balb/c* mice with low-dose ME49^WT^ and ME49^Δ*gra35*^ tachyzoites. (e) Survival curves after infection of *Balb/c* mice with high-dose ME49^WT^ and ME49^Δ*gra35*^ tachyzoites. (f) Comparison of neuronal damage in mouse brain between ME49^WT^ and ME49^Δ*gra35*^ by NeuN and Cleaved Caspase-3 immunofluorescence staining. UI, uninfected. Thirty 6–8 weeks old C57BL/6 female mice were randomly divided into PBS, ME49^WT^, and ME49^Δ*gra35*^ groups. Each group was injected intraperitoneally with 1 × 10^3^ *Toxoplasma gondii* per mouse. 2 months later, each mouse was injected intraperitoneally with cyclophosphamide (50 mg/kg) to induce recurrence of toxoplasmosis. 7 days later, all mice were euthanized, and brain tissues were removed and analyzed. NeuN (red), Cleaved Caspase-3 (green) and DAPI (blue); scale bar = 50 μm. Each bar represents the mean ± SD (*n* = 3). ***p < 0.001; **p < 0.01; *p < 0.05; ns, not significant.

We conducted phenotypic analysis on the ME49^Δ*gra35*^ strain of parasites. The Giemsa staining results revealed a significantly higher proliferation rate in the ME49^Δ*gra35*^ group compared to the ME49^WT^ group (Figure 2(a,b)). We further examined cyst formation using staining with FITC-labeled Dolichos Biflorus Agglutinin (DBA). Brain cyst counting revealed a significant reduction in cyst numbers in C57BL/6 mice infected with ME49^Δ*gra35*^ compared to ME49^WT^ (Figure 2(c)). Upon infecting C57BL/6 mice with low doses of both ME49 strains (1 × 10^2^ parasites), survival rates exceeded 30 days without significant differences between the two groups (Figure 2(d)). In contrast, infection with a high dose of ME49^WT^ (1 × 10^6^ parasites) resulted in nearly 100% mortality within 10 days, while infection with ME49^Δ*gra35*^ extended the survival time by up to 20 days (Figure 2(e)). We used Immunofluorescent assay staining with NeuN (a specific neuron marker) and Cleaved Caspase-3 to detect the neuronal apoptosis during TE. The C57BL/6 mice was injected intraperitoneally with 1 × 10^3^ *Toxoplasma gondii* and continued to be reared for 2 months. To induce an acute exacerbation of chronic *Toxoplasma* infection, each mouse received a daily intraperitoneal injection of 50 mg/kg cyclophosphamide for seven consecutive days. Mice were euthanized, brain tissue was collected, fixed, embedded, sectioned and stained. The results demonstrated co-localization of Cleaved Caspase-3 with the neuronal marker NeuN in the cerebral cortex, and ME49^WT^-infected mice exhibited a more pronounced occurrence of neuronal apoptosis in the cerebral cortex compared to those infected with ME49^Δ*gra35*^ (Figure 2(f)). Therefore, GRA35 was a key factor to induce neuronal apoptosis during toxoplasmosis.

### GRA35 played a facilitating role in neuronal apoptosis in vitro

**Figure 3.**
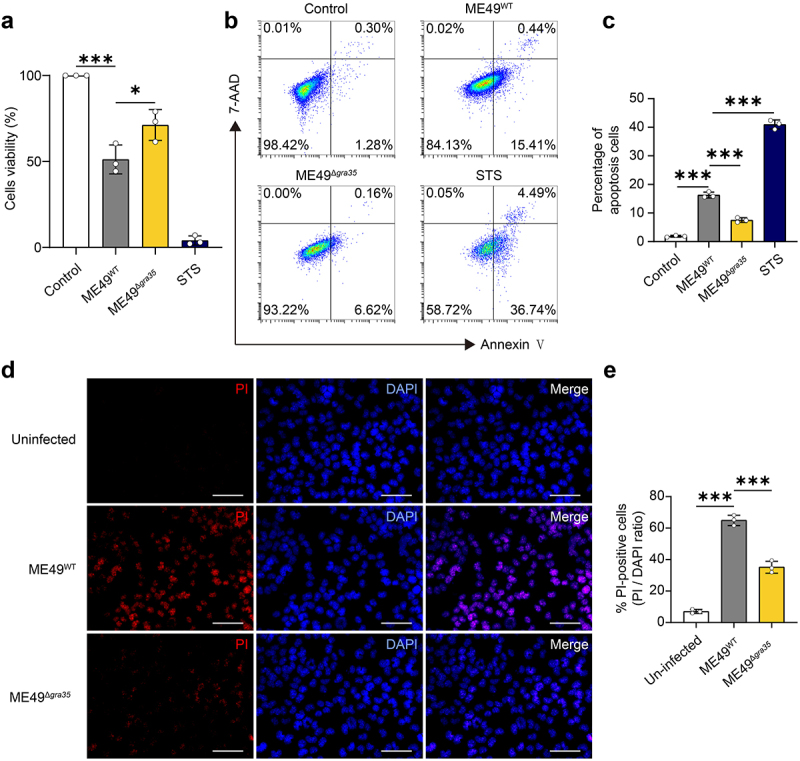
GRA35 elicited neuronal apoptosis and reduced cellular viability *in vitro*. (a) cell viability was evaluated by CCK8 after 24 hours of tachyzoites invasion. The cell viability (%) was calculated as: the absorbance of treatment group cells/the absorbance of control group cells × 100%. STS is staurosporine, an inducer of apoptosis. (b) Differences in the effects of ME49^WT^ and ME49^Δ*gra35*^ on the apoptosis of retinoic acid (RA)-induced Neuro2a cells were detected by flow cytometry. (c) Statistical results of (b). (d) Differences in the effects of ME49^WT^ and ME49^Δ*gra35*^ on the apoptosis of RA-induced Neuro2a cells were detected by PI stain; (scale bar = 20 μm). (e) Quantification of PI-positive cells in (d). Each bar represents the mean ± SD (*n* = 3). ***p < 0.001; **p < 0.01; *p < 0.05; ns, not significant.

To investigate the impact of GRA35 on neuronal cells *in vitro*, we utilized the Neuro2a cells line. Based on the fact that Neuro2a cells are neural crest derived cells that need to be differentiated to neurons, we treated Neuro2a cells with retinoic acid (RA, 20 μM) to induce neural differentiation [20–22]. Then we infected these neuronal cells with ME49^WT^ and ME49^Δ*gra35*^ strains at MOI of 3 for 24 hours. The CCK8 assay indicated that cell viability was significantly inhibited following ME49^WT^ infection, which was attenuated by GRA35 knockout (Figure 3(a)). Flow cytometry results indicated that GRA35 facilitated neuronal apoptosis (Figure 3(b, c)). The PI staining results showed a higher proportion of cell death infected with ME49^WT^ (Figure 3(d, e)). These results suggested that GRA35 elicited neuronal apoptosis and reduced cellular viability *in vitro*.

### GRA35 induces a decrease in mitochondrial membrane potential and permeability

**Figure 4.**
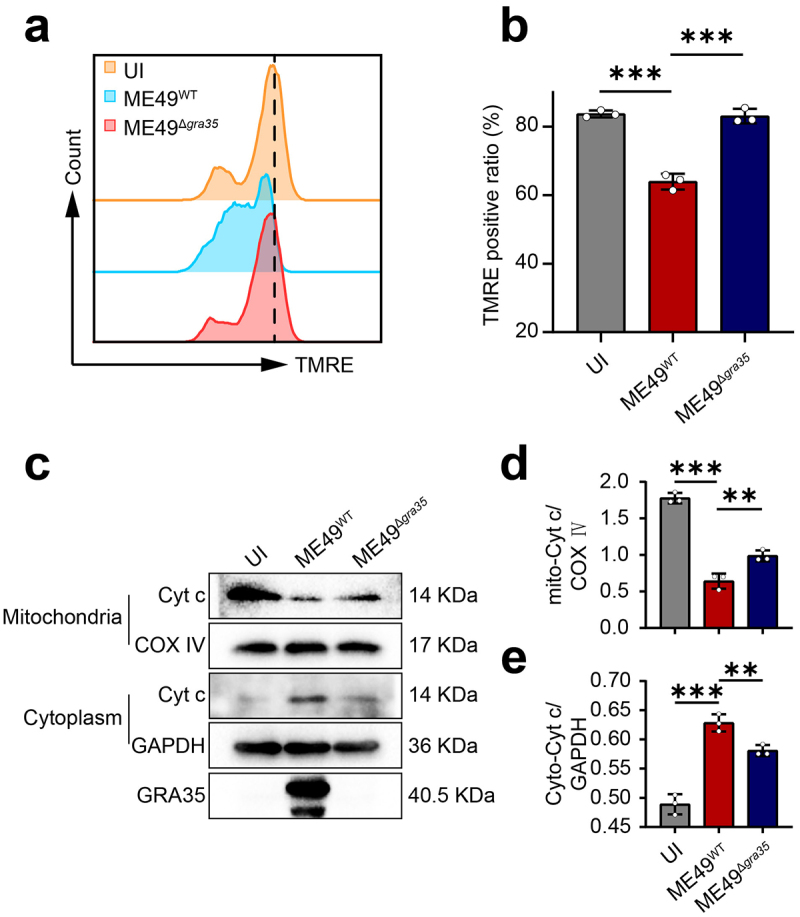
GRA35 induced a decrease in mitochondrial membrane potential and permeability. (a) Mitochondrial membrane permeability was detected by flow cytometry. 1 × 10^6^ Neuro2a cells were seeded into each well of a 6-well plate, 3 × 10^6^ ME49^WT^, ME49^Δ*gra35*^ tachyzoites were added after 24 h of culture, respectively, continued to be cultured for 24 h until detection. (b) Statistical results of (a). (c) Effect of ME49^WT^ and ME49^Δ*gra35*^ on mitochondrial membrane permeability detected by WB. 1 × 10^6^ RA-induced Neuro2a cells were seeded into each well of a 6-well plate, 3 × 10^6^ ME49^WT^, ME49^Δ*gra35*^ tachyzoites were added after 24 h of culture, respectively, continued to be cultured for 24 h until detection. (d) Statistical analysis results for mitochondrial cytochrome c shown in panel (c). (e) Statistical analysis results for cytoplasmic cytochrome c shown in panel (c). Each bar represents the mean ± SD (*n* = 3). ***p < 0.001; **p < 0.01; *p < 0.05; ns, not significant.

To investigate the apoptotic mechanism triggered by *T. gondii* GRA35, we assessed the mitochondrial membrane potential in neuronal cells 24 hours post-infection with ME49^WT^ and ME49^Δ*gra35*^ strains (MOI = 3). Our findings revealed a significant decrease in mitochondrial membrane potential following infection with ME49^WT^. Whereas ME49^Δ*gra35*^ infection led to a notable increase in mitochondrial membrane potential compared to the ME49^WT^ (Figure 4(a,b)). Subsequently, we evaluated mitochondrial permeability of cells following 24 hours of infection (MOI = 3) with ME49^WT^ and ME49^Δ*gra35*^ strains. The findings revealed a significant reduction in mitochondrial cytochrome c levels in ME49^WT^-infected cells compared to the ME49^Δ*gra35*^-infected cells. Conversely, ME49^WT^-infected cells exhibited a significant elevation in cytoplasmic cytochrome c content compared to the ME49^Δ*gra35*^-infected cells (Figure 4(c-e)). These findings suggested that GRA35 facilitated the reduction of mitochondrial membrane potential and enhanced mitochondrial permeability in infected neuronal cells.

### GRA35 directly interacts with the host RTN1-c

**Figure 5.**
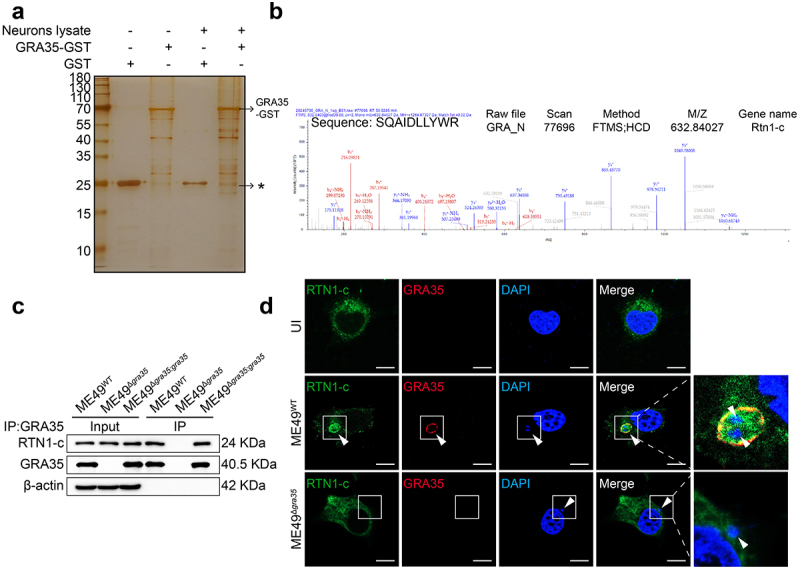
GRA35 was capable of interacting with RTN1-c. (a) GST-Pull down silver staining results. The lysed neuronal cells were incubated with GST-beads and GST-GRA35-beads to detect all proteins that might interact with GRA35. The asterisk denotes the RTN1c protein band. (b) Mass spectrum identification map of RTN1-c. (c) IP assay to detect the binding of GRA35 to RTN1-c. Neuro2a cells were respectively infected with ME49^WT^,ME49^Δ*gra35*^ and ME49^Δ*gra35:gra35*^ strains for 24 hours. Input and immunoprecipitates were analyzed by WB. (d) Co-staining of RTN1-c with GRA35. The white box magnifies the *Toxoplasma gondii* in the cell. And the white arrow highlights the *Toxoplasma gondii* in the infected neuron cells. Scale bar = 10 μm. The RA-induced Neuro2a cells were infected with ME49^WT^ and ME49^Δ*gra35*^ tachyzoites, respectively (MOI = 3), for 24 hours. Subsequently, the cells were double stained with RTN1-c (green) and GRA35 (red).

To elucidate the underlying mechanism of GRA35-induced modulation in mitochondrial membrane potential and permeability, we performed a GST pull-down assay. Initially, a pGEX-6p-1-GRA35 plasmid was constructed, leading to the expression of rGRA35-GST protein. Subsequently, the GST pull-down assay was utilized to identify proteins that interact with GRA35 in neuronal cells (Figure 5(a)). We excised the entire lane of the SDS-PAGE gel for mass spectrometry analysis. RTN1-c, a protein mostly found in neurons, captured our attention (Figure 5(a,b)). To validate the results obtained from mass spectrometry, we designed a pCMV-Myc-GRA35-HA plasmid (Figure S1a-c) for conducting Co-IP experiments. The interaction between GRA35 and RTN1-c was confirmed through a Co-IP experiment (Figure S2a). Moreover, the immunofluorescence experiment of the pCMV-myc-GRA35-HA cell line also demonstrated that GRA35 and RTN1-c have co-localization (Figure S2b). Subsequently, neuronal cells were infected with ME49^WT^, ME49^Δ*gra35*^, and ME49^Δ*gra35:gra35*^ strains, and Co-IP assays were performed to investigate the interaction between endogenous *Toxoplasma* GRA35 and neuronal RTN1c (Figure 5(c)). Furthermore, immunofluorescence assay demonstrated the co-localization of *Toxoplasma* ME49 strain GRA35 with RTN1-c in parasites-infected neuronal cells (Figure 5(d)). Thus, these results supported that *Toxoplasma* GRA35 directly interacted with the host RTN1-c during infection.

### GRA35 induces mitochondrial apoptosis by displacing Bcl-XL from mitochondria through its interaction with RTN1-c

**Figure 6.**
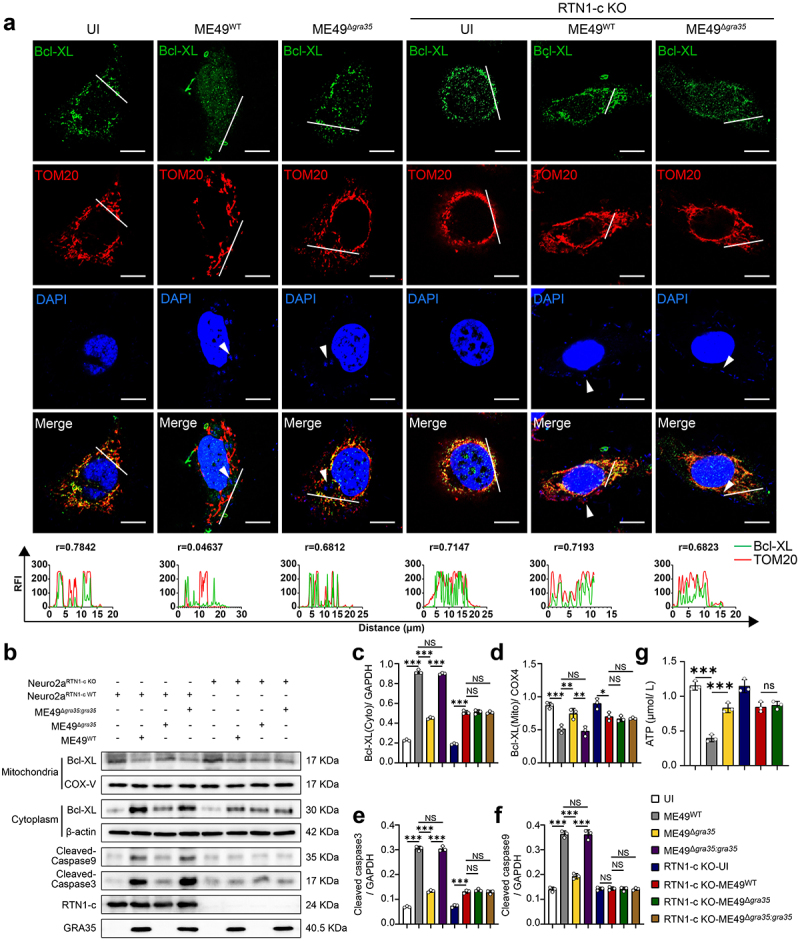
GRA35 triggered mitochondrial apoptosis by binding to RTN1-c and reducing the distribution of Bcl-XL in mitochondria. (a) Co-staining of Bcl-XL with TOM20. The white arrow indicates the *Toxoplasma gondii* in the infected neuron cells. Scale bar = 10 μm. The RA-induced Neuro2a cells were infected with ME49^WT^ and ME49^Δ*gra35*^ tachyzoites, respectively (MOI = 3), for 24 hours. Subsequently, the cells were double stained with Bcl-XL (green) and TOM20 (red). TOM20 is widely acknowledged as the mitochondrial protein. The intensity profiles of the green channel and the red channel in each group was shown in the lower panel. Colocalization analysis was conducted by calculating the Pearson correlation coefficient (r value) and assessing relative fluorescence intensity (RFI) along the specified trajectories. (b) WB analysis was used to determine the expression of proteins related to apoptosis. (c-f) Statistical results of (b). COX-657IV is widely acknowledged as the mitochondrial protein. (g) Quantification of ME49^WT^ and ME49^Δ*gra35*^ on ATP activity in Neuro2a cells. Each bar represents the mean ± SD (*n* = 3). ***p < 0.001; **p < 0.01; *p < 0.05; ns, not significant.

RTN1-c has been reported to bind to Bcl-XL, which affects its translocation into mitochondria and promotes cell apoptosis through the mitochondrial pathway [27]. Given the interaction between GRA35 and RTN1-c, we further investigated the role of GRA35 in the mitochondrial apoptosis pathway. Immunofluorescence assay showed a disruption in the co-localization of Bcl-XL and mitochondria in neuronal cells infected with the ME49^WT^ strain, whereas re-localization of Bcl-XL with mitochondria was observed following infection with the ME49^Δ*gra35*^ strain (Figure 6(a)). The results suggested that GRA35 and RTN1-c conjunction reduced the distribution of Bcl-XL in mitochondria (Figure 6(a)). Consistently, subcellular localization studies using Western blotting revealed that neuronal cells infected with the ME49^WT^ strain exhibited the lowest mitochondrial expression of Bcl-XL. Conversely, infection with either the ME49^Δ*gra35*^ strain or RTN1-c knockout resulted in increased levels of Bcl-XL within mitochondria compared to the ME49^WT^ infection group (Figure 6(b-d)). Furthermore, Cleaved Caspase-3 levels were significantly higher in neuronal cells infected with the ME49^WT^ strain compared to other groups. Notably, infection with the ME49^Δ*gra35*^ strain or knockout of RTN1-c resulted in a lower expression of Cleaved Caspase-3 and Cleaved Caspase-9 when compared to the ME49^WT^ infection group, indicating that GRA35 triggered mitochondrial apoptosis by binding to RTN1-c and reducing the distribution of Bcl-XL in mitochondria (Figure 6(b, e, f)). ATP levels typically decrease alongside a reduction in the mitochondrial membrane potential during cellular apoptosis. Thus, we used ATP activity assay to evaluate the effect of GRA35 on mitochondrial function. The result suggested that cells infected with the ME49^WT^ strain exhibited significantly lower ATP activity compared to those infected with the ME49^Δ*gra35*^ strain (Figure 6(g)). In contrast, RTN1-c knockout neuronal cells almost abolished this decrease, suggesting that the RTN1-c is essential for GRA35-induced apoptosis by mitochondrial pathway (Figure 6(g)). In conclusion, these results suggested that GRA35 induced mitochondrial apoptosis by preventing Bcl-XL from entering mitochondria through its interaction with RTN1-c.

### GRA35-induced neuronal apoptosis does not solely dependent on RTN1-c

**Figure 7.**
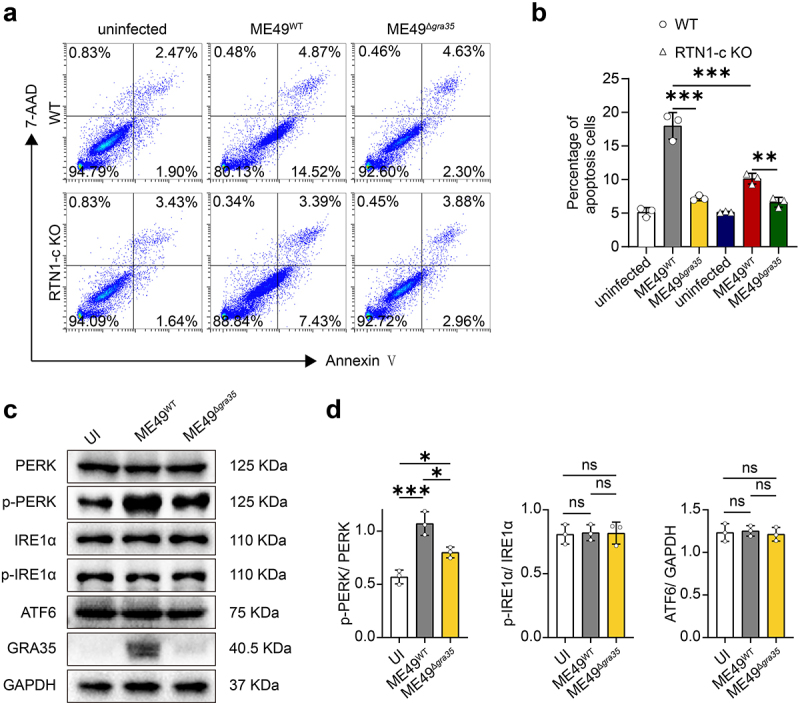
GRA35-induced neuronal apoptosis does not solely dependent on RTN1-c.

To examine whether GRA35-induced neuronal apoptosis is dependent on RTN1-c, we used flow cytometry assay and found the partial reversal of the pro-apoptotic effect of GRA35 following RTN1-c knockout suggesting that there are other signaling pathways contributing to neuronal apoptosis besides the GRA35- RTN1-c mechanism (Figure 7(a,b)). It is known that apoptotic pathways include the death receptor pathway, mitochondrial pathway, and ER pathway [9–11]. As noted above, GRA35 was localized on the *Toxoplasma* parasitophorous vacuole membrane (PVM), while receptors involved in the death receptor pathway are found on the extracellular membrane [12,19]. Thus, it is unlikely that GRA35 activates the host cell’s death receptor pathway, and the analysis of Caspase-8 also confirmed our hypothesis (Figure S3a, b). Therefore, we investigated whether the presence of GRA35 would elicit ER stress. We conducted preliminary validation using Western blot experiments and observed that GRA35 induces activation of the PERK pathway in ER stress, while the IRE1 and ATF6 pathways remain un-activated (Figure 7(c, d)).

(a) The apoptosis level of neurons infected with ME49^WT^ and ME49^Δ*gra35*^ after RTN1-c knockout was assessed using flow cytometry. (b) Quantification of (a). (c) Effect of ME49^WT^ and ME49^Δ*gra35*^ on ER stress-related proteins in RA-induced Neuro2a cells detected by WB assay. The RA-induced Neuro2a cells were infected with ME49^WT^ and ME49^Δ*gra35*^ tachyzoites, respectively (MOI = 3), for 24 hours until detection. (d) Statistics of ER stress-related proteins in **c**. Each bar represents the mean ± SD (*n* = 3). ***p < 0.001; **p < 0.01; *p < 0.05; ns, not significant.

### GRA35 induces ER stress associated apoptosis via

**Figure 8.**
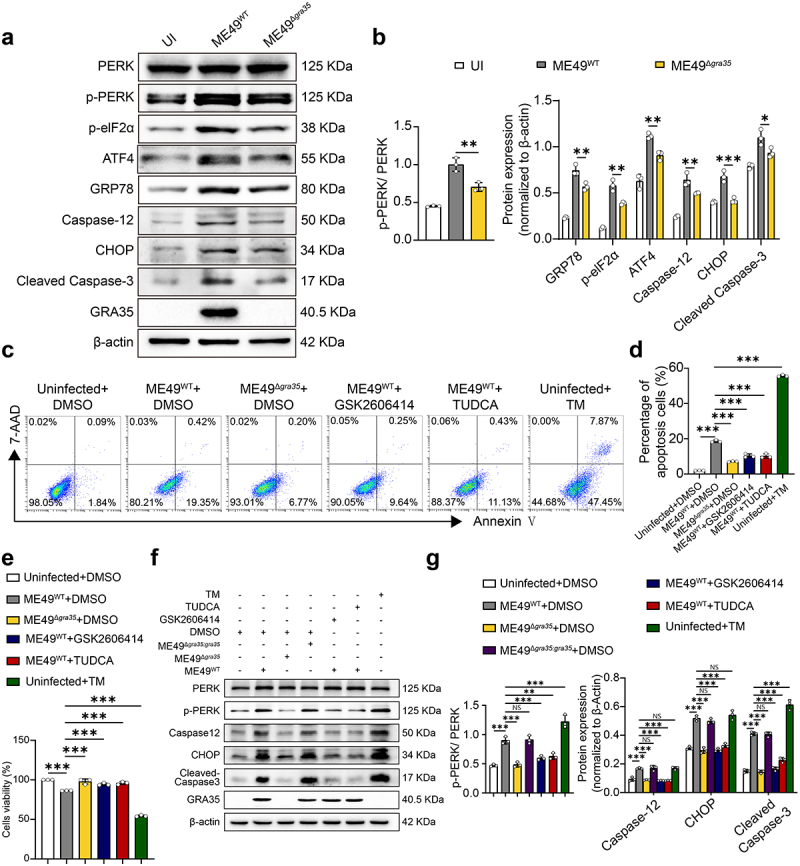
GRA35 induced er stress-mediated apoptosis through perk branch in neurons.

For the purpose of further substantiation, relevant experiments were conducted to further validate the PERK pathway in ER stress. The results revealed that the ME49^WT^ infection group exhibited elevated levels of phosphorylated PERK and elF2α, along with increased expression of ATF4, GRP78, CHOP, Caspase-12 and Cleaved Caspase-3, compared to the ME49^Δ*gra35*^ infection group, suggesting that GRA35 induces ER stress-associated apoptosis through PERK branch (Figure 8(a,b)). Additionally, tunicamycin (TM), an ER stress inducer, exhibited the highest apoptosis rate. ME49^Δ*gra35*^ infection group exhibited the lowest apoptosis rate compared to the ME49^WT^ infection group. GSK2606414, a potent inhibitor of PERK, inhibited the level of apoptosis induced by GRA35. TUDCA, a well-established inhibitor of ER stress, also greatly inhibited the level of apoptosis induced by GRA35 (Figure 8(c,d)). The CCK8 result also revealed that ME49^Δ*gra35*^ infection group significantly enhanced cell viability compared to the ME49^WT^ infection group. However, the addition of GSK2606414 to the ME49^WT^ group improved the viability (Figure 8(e)). Consistently, western blot analysis revealed that ME49^WT^ group greatly decreased CHOP, Caspase-12, Cleaved Caspase-3 and the phosphorylation of PERK in neuronal cells after GSK2606414 stimulation (Figure 8(f,g)). Additionally, over-expressed pCMV-Myc-GRA35-HA also confirmed the above results (Figure S4a-e). Altogether, these data confirmed that GRA35 induced ER stress associated neuronal apoptosis *via* the PERK signaling pathway.

(a) Effect of ME49^WT^ and ME49^Δ*gra35*^ on ER stress-related proteins in RA-induced Neuro2a cells detected by WB assay (*n* = 3). The Neuro2a cells were infected with ME49^WT^ and ME49^Δ*gra35*^ tachyzoites, respectively (MOI = 3), for 24 hours until detection. (b) Quantification of (a). (c) Differences in the effects of ME49^WT^ and ME49^Δ*gra35*^ on RA-induced Neuro2a cells apoptosis detected by flow cytometry(*n* = 3). (d) Quantification of (c). (e) Differences in the effects of ME49^WT^ and ME49^Δ*gra35*^ on RA-induced Neuro2a cells activity detected by CCK8 assay. (f) Effect of ME49^WT^, ME49^Δ*gra35*^ and ME49^Δ*gra35:gra35*^ on ER stress pathway in Neuro2a cells detected by WB. The RA-induced Neuro2a cells were infected with ME49^WT^, ME49^Δ*gra35*^ and ME49^Δ*gra35:gra35*^ tachyzoites, respectively (MOI = 3), for 24 hours. GSK2606414 is a PERK inhibitor; TUDCA is an ER stress inhibitor; TM is tunicamycin, an ER stress activator. (g) Quantification of (f). Each bar represents the mean ± SD (*n* = 3). ***p < 0.001; **p < 0.01; *p < 0.05; ns, not significant.

## Discussion

*T. gondii* has many genetically distinct strain types, with the most studied strains being the canonical strains called type I, II, and III [28,29]. Type II *Toxoplasma gondii* represents one of the predominant strains implicated in the establishment of chronic infections in humans, characterized by its ability to maintain a latent state within host tissues, including brain and muscle tissue [26,30,31]. Nonetheless, if an individual’s immune system is significantly compromised-especially in the context of conditions such as HIV/AIDS, organ transplantation, or immunosuppressive therapy, the latent *Toxoplasma gondii* infection has the potential to reactivate, resulting in active infection with the risk of severe clinical manifestations, including TE [4,32,33]. Within the life cycle of *Toxoplasma gondii*, the GRA proteins serve as critical effector molecules involved in vacuole remodeling, nutrient uptake, and evasion of host immune responses [34]. Studies have indicated that certain GRAs have the ability to impact host cell functions by crossing the parasitophorous vacuole membrane [34]. GRA15 is capable of being secreted into host cells to regulate the nuclear translocation of NF-κB transcription factor, thereby activating the NF-κB signaling pathway to promote M1 polarization of host macrophages [35]. GRA24 has been shown to enter host cell nuclei and induce prolonged nuclear translocation response through self-phosphorylation of p38α MAP kinase [36].

GRA35 is a recently identified effector protein of *Toxoplasma gondii*, which is located on the parasitophorous vacuole membrane (PVM). Current research only indicates that it is a key effector molecule for inducing NLRP1-dependent pyroptosis in Lewis rat macrophages [19]. Additionally, GRA35 can promote the recruitment of RNF213 to the PVM in IFN-γ-primed human fibroblasts (HFF) through the E3 ubiquitin ligase ITCH, thereby driving K63-linked ubiquitination modification of the PVM and inhibiting *Toxoplasma gondii* replication [37]. However, studies on the function of GRA35 are still very limited, especially in the central nervous system, which have not been reported to date.

In this study, we identified a significant upregulation of the GRA35 gene in less virulent strains of *Toxoplasma gondii*. Utilizing CRISPR-Cas9 technology, we genetically disrupted the expression of the GRA35 gene in the ME49 strain of *T. gondii* and extensively investigated its function. Our findings revealed that mice infected with ME49^Δ*gra35*^ parasites exhibited markedly reduced mortality rates, accompanied by a diminished ability of these parasites to form cysts within brain tissue. Interestingly, the ME49^Δ*gra35*^ parasites exhibited enhanced proliferative capacity compared to the ME49^WT^ strain *in vitro*. Further investigation in an acute mouse model of cerebral toxoplasmosis revealed that GRA35 plays a pivotal role in promoting neuronal cell apoptosis.

**Figure 9.**
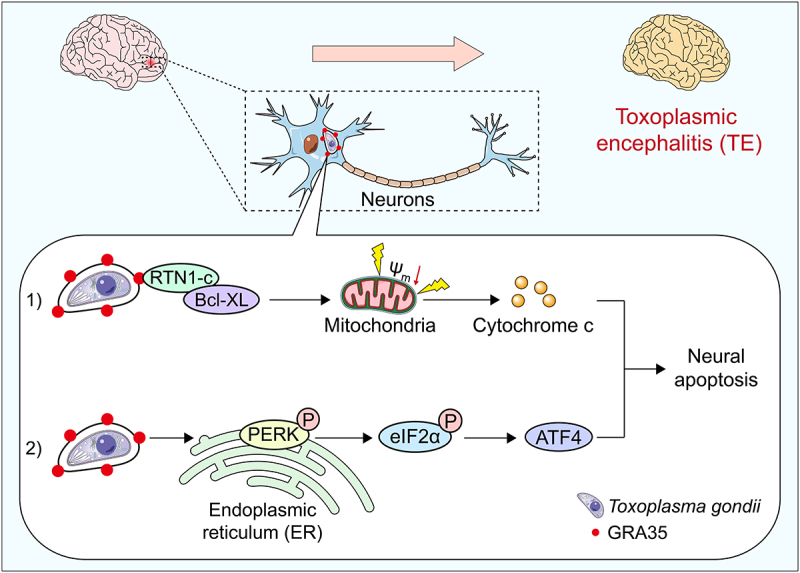
*Toxoplasma gondii* effector GRA35 mediated brain damage via er stress and mitochondria-associated apoptosis.

Neuronal apoptosis plays a significant role in the pathogenesis of TE, with inflammation identified as a key pathway leading to neuronal cell death [8]. Recent research has shown that *Toxoplasma* proteins directly damage neurons, contributing to their demise [38]. A study utilizing a mouse model and a customized MATLAB mapping program revealed abnormal electrophysiological activity in neurons injected with *Toxoplasma* proteins, resulting in an approximately 90% mortality rate within 8 weeks [38]. Herein, this investigation utilized Cleaved Caspase-3 and NeuN co-labeling techniques to confirm the ability of the *Toxoplasma* effector GRA35 to promote neuronal apoptosis. GST-pull down experiments exploring potential mechanisms revealed that GRA35 can bind to the neuron-specific molecule RTN1-c. Previous study suggests that the central nervous system-specific protein RTN1-c interacts with Bcl-XL to prevent its translocation into mitochondria, thereby facilitating mitochondria-mediated apoptotic processes [27,39]. Our further study indicated that GRA35 accelerates neuronal apoptosis by formation the GRA35-RTN1-c-Bcl-XL complex and impeding Bcl-XL’s mitochondrial translocation. However, knockout of RTN1-c did not fully restore the apoptosis rate of ME49^WT^ cells to the level observed in ME49^Δ*gra35*^ strain, suggesting that GRA35 May promote host cell apoptosis through alternative pathways. Previous studies have demonstrated that *Toxoplasma* effector molecules such as ROP18 and GRA3, can induce apoptosis by inducing ER stress in host cells [7,8]. Therefore, we investigated whether GRA35 could elicit ER stress in neuronal cells. Experimental evidence using a combination of PERK inhibitor and ER stress inhibitor revealed that GRA35 can indeed induce ER stress *via* the PERK pathway. Consequently, GRA35 can directly facilitate apoptosis in neuronal cells through both mitochondrial apoptotic signaling and ER stress-associated apoptosis (Figure 9).

The *Toxoplasma gondii* protein GRA35 located on the parasitophorous vacuole membrane induces neuronal cell apoptosis *via* two distinct mechanisms: 1) GRA35 binding with RTN1-c to inhibit the entry of Bcl-XL into mitochondria, resulting in the decline of mitochondrial membrane potential and the release of cytochrome c from mitochondria, thus accelerating neural apoptosis. 2) GRA35 phosphorylated PERK in the ER stress pathway and promotes ER stress related apoptosis. Eventually, these two apoptotic pathways act on neuron and promote the development of TE.

In this study, we discovered that GRA35 in *Toxoplasma gondii* tachyzoites can induce the apoptosis of neuronal cells. Nevertheless, it is still unclear whether GRA35 in *Toxoplasma gondii* bradyzoites has the same effect. Research indicates that the GRA35 protein is present in the cyst wall during chronic *Toxoplasma gondii* infection [40]. Therefore, during chronic infection, GRA35 might interact with host proteins, thus promoting the apoptosis of neuronal cells. Notably, GRA35 knockout significantly reduced the number of brain cysts in infected mice, indicating that GRA35 plays a critical role in cyst persistence within the central nervous system and highlighting its potential as a therapeutic target for toxoplasmic encephalitis. In subsequent studies, we will conduct further explorations of the role of the GRA35 protein during chronic infection.

Our research reveals the significant contribution of GRA35 to the pathogenesis of toxoplasmosis, enhancing our understanding and management of this disease. Nevertheless, more research is necessary for future investigations. For example, the investigation of neuronal cells in complex environments in mice and humans requires more than just focusing on stimulating neuronal apoptosis. It is essential to explore how the release of GRA35 triggers host neuronal necroptosis. In cases of TE, the presence of immune cells at lesion sites initiates cascading reactions that lead to significant damage. However, it remains unclear whether GRA35 can attract immune cells and, if so, which specific types are recruited. In summary, GRA35 mediates ER stress and mitochondria-associated apoptosis, and serves as a crucial virulence factor in the pathogenesis of TE.

## Conclusions

The *Toxoplasma gondii* protein GRA35 located on the parasitophorous vacuole membrane induces neuronal cell apoptosis. On the one hand, GRA35 conjuncted with RTN1-c to inhibit the entry of BCL-XL into mitochondria, resulting in the decline of mitochondrial membrane potential and the release of cytochrome c from mitochondria, thus accelerating neural apoptosis. On the other hand, GRA35 phosphorylated PERK in the ER stress pathway and promotes ER stress related apoptosis. Eventually, these two apoptotic pathways act on neuron and promote the development of TE.

## Supplementary Material

Supplementary Material.doc

## Data Availability

The mass spectrometry raw data of Figure 5 are openly available in the ScienceDB (https://doi.org/10.57760/sciencedb.29128 [41]) and in the ProteomeXchange database at https://www.ebi.ac.uk/pride/archive/projects/PXD067653, accession number PXD067653. The data that support the findings of this study are openly available in figshare at (http://doi.org/10.6084/m9.figshare.29329469 [42]).

## ARRIVE statement

In this study, all experiments involving live animals were conducted in accordance with the ARRIVE guidelines. And the ARRIVE checklist was uploaded as a supplementary file.
