# Supplementary material for: Toxoplasma gondii effector GRA35 mediates neuronal damage via ER stress and mitochondria-associated apoptosis
Source: Virulence. 2026 Apr 6;17(1):2654261. doi: 10.1080/21505594.2026.2654261 (PMC13078242; doi:10.1080/21505594.2026.2654261)
Supplement: Supplementary Material.doc [file KVIR_A_2654261_SM9544.doc]

**Supplemental data**


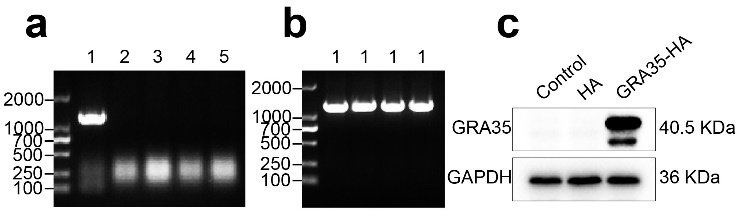


**Figure S1.** pCMV-Myc-GRA35-HA plasmid construction. (A) PCR validation of the GRA35-HA fragment in different colony. The numbers 1-5 represent five different colonies. (B) PCR validation of the pCMV-Myc-GRA35-HA plasmid extracted from colony 1 in (A). (C) WB validation of pCMV-Myc-GRA35-HA plasmid.


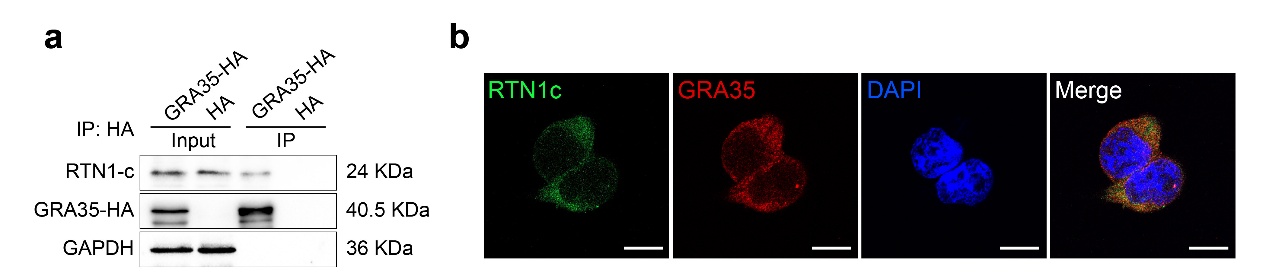


**Figure S2.** GRA35 is able to interact with RTN1c. (A)IP assay to detect the binding of GRA35 to RTN1-c. Neuro2a cells were transfected with pCMV-Myc-GRA35-HA or control vector for 24 h. Input and immunoprecipitates were analyzed by WB. (B) Co-staining of RTN1-c with GRA35. Scale bar=10 μm. The RA-induced Neuro2a cells were transfected with pCMV-Myc-GRA35-HA for 24 h. Subsequently, the cells were double stained with RTN1-c (green) and GRA35 (red).


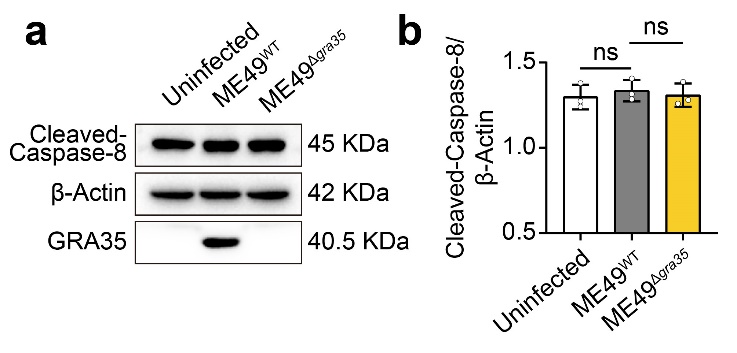


**Figure S3.** GRA35 does not facilitated extrinsic apoptosis. (A) The extrinsic apoptosis level of neurons infected with ME49^WT^ and ME49^Δ^*^gra35^* was assessed using WB. (B) Quantification of (A), each bar represents the mean ± SD (n = 3). ****p* < 0.001; ***p* < 0.01; **p* < 0.05; ns, not significant.


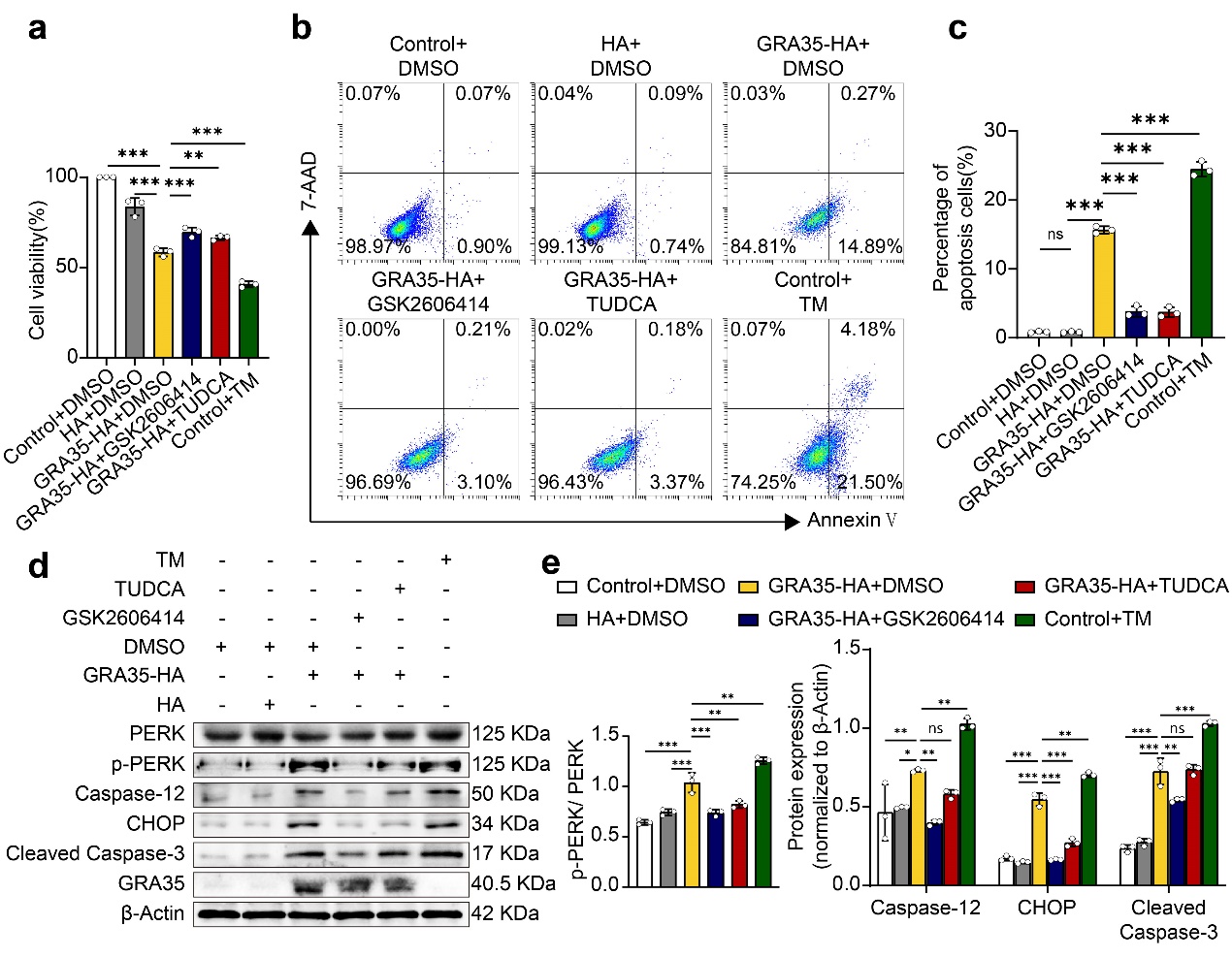


**Figure S4.** Transfection of pCMV-Myc-GRA35-HA plasmid activates ER stress and its related apoptosis in neurons. (A) Effects of transfection of pCMV-Myc-GRA35-HA plasmid on neuronal cells activity detected by CCK8 assay. (B) Effects of transfection of pCMV-Myc-GRA35-HA plasmid on neurons apoptosis detected by flow cytometry. (C) Statistical results of (B). (D) Effect of transfection of pCMV-Myc-GRA35-HA plasmid on ER stress and its related apoptosis in neurons detected by WB. (E) Statistical results of (D). Each bar represents the mean ± SD (n = 3). ****p* < 0.001; ***p* < 0.01; **p* < 0.05; ns, not significant.

**Supplementary Table S1.** Specific primers for GRA35 knockout ME49 strains construction.

| Primers | Sequence (5’-3’) |
| --- | --- |
| SgGRA35-F | ACGTGCTACTGGAAAAACAGGTTTTAGAGCTAGAAATAGC |
| SgGRA35-R | AACTTGACATCCCCATTTAC |
| GRA35UP-F | AAAACGACGGCCAGTGAATTCACTCGATAACATGCAGCCTCATC |
| GRA35UP-R | CGGGGGGTGAAAATCGAATGACAGGTTGTCCAAAGAGTTTTTTTCTAGTCTC |
| GRA35DW-F | CTATGCACTTGCAGGATGAATTCCTGACGTCTTCGAACAGTTGAGGA |
| GRA35DW-R | GACCATGATTACGCCAAGCTTACCCATGATTACGTTGGCGA |
| DHFR-F | AAAAAACTCTTTGGACAACCTGTCATTCGATTTTCACCCCCCG |
| DHFR-R | CAACTGTTCGAAGACGTCAGGAATTCATCCTGCAAGTGCATAG |
| PUC19-F | GAATTCACTGGCCGTCGTTTT |
| PUC19-R | AAGCTTGGCGTAATCATGGTC |
| PCR1-F | GGTGAATCATAATTGTGGGCCCC |
| PCR1-R | GACAACGAATGACACACAGGAACTAC |
| PCR2-F | GACAGCAGACAACTTTCCTTCTATGC |
| PCR2-R | CCACTTCGGGAGTTTACGTGTTA |
| PCR3-F | TGGGTGAGTCATGACATCACGT |
| PCR3-R | ACCTAGATACGTGAAGAATAAGCCACA |

The primers SgGRA35-F and SgGRA35-R are used to construct the plasmid pSAG1::CAS9-U6::SgGRA35; The primers GRA35UP-F, GRA35UP-R, GRA35DW-F, GRA35DW-R, DHFR-F, DHFR-R, PUC19-F, and PUC19-R are used to construct the donor fragment spanning from GRA35-up to GRA35-down; The primers PCR1-F and PCR1-R demonstrate successful integration of upstream homology arms of GRA35; The primers PCR2-F and PCR2-R demonstrate successful integration of downstream homology arms of GRA35; The primers PCR3-F and PCR3-R demonstrate successful knockout of the *gra35* gene.

**Supplementary Table S2. Specific primers for pCMV-Myc-GRA35-HA plasmid construction.**

| Primers | Sequence (5’-3’) |
| --- | --- |
| ECOR1-GRA35-HA-F | CCGGAATTCGGATGTATCCCCTGACTGTTTACTCGAT |
| XHO1-GRA35-HA-R | CCGCTCGAGTCAAGTCTGTTTCGGTTCCGC |
